# Altered m6A RNA methylation governs denervation-induced muscle atrophy by regulating ubiquitin proteasome pathway

**DOI:** 10.1186/s12967-023-04694-3

**Published:** 2023-11-23

**Authors:** Junjie Sun, Hai Zhou, Zehao Chen, Han Zhang, Yanzhe Cao, Xinlei Yao, Xin Chen, Boya Liu, Zihui Gao, Yuntian Shen, Lei Qi, Hualin Sun

**Affiliations:** 1 Key Laboratory of Neuroregeneration of Jiangsu and Ministry of Education, Co-Innovation Center of Neuroregeneration, NMPA Key Laboratory for Research and Evaluation of Tissue Engineering Technology Products, Nantong University, Nantong, 226001 Jiangsu People’s Republic of China; 2 Department of Neurosurgery, Binhai County People’s Hospital, Yancheng, 224500 Jiangsu People’s Republic of China; 3 Department of Clinical Medicine, Medical College, Nantong University, Nantong, 226001 China; 4 Department of Neurology, Affiliated Hospital of Nantong University, Nantong, 226001 Jiangsu People’s Republic of China; 5 Department of Emergency Medicine, Affiliated Hospital of Nantong University, Nantong, 226001 Jiangsu People’s Republic of China

**Keywords:** Muscle atrophy, Denervation, m6A, Ubiquitin–proteasome pathway

## Abstract

**Background:**

Denervation-induced muscle atrophy is complex disease involving multiple biological processes with unknown mechanisms. N6-methyladenosine (m6A) participates in skeletal muscle physiology by regulating multiple levels of RNA metabolism, but its impact on denervation-induced muscle atrophy is still unclear. Here, we aimed to explore the changes, functions, and molecular mechanisms of m6A RNA methylation during denervation-induced muscle atrophy.

**Methods:**

During denervation-induced muscle atrophy, the m6A immunoprecipitation sequencing (MeRIP-seq) as well as enzyme-linked immunosorbent assay analysis were used to detect the changes of m6A modified RNAs and the involved biological processes. 3-deazidenosine (Daa) and R-2-hydroxyglutarate (R-2HG) were used to verify the roles of m6A RNA methylation. Through bioinformatics analysis combined with experimental verification, the regulatory roles and mechanisms of m6A RNA methylation had been explored.

**Results:**

There were many m6A modified RNAs with differences during denervation-induced muscle atrophy, and overall, they were mainly downregulated. After 72 h of denervation, the biological processes involved in the altered mRNA with m6A modification were mainly related to zinc ion binding, ubiquitin protein ligase activity, ATP binding and sequence-specific DNA binding and transcription coactivator activity. Daa reduced overall m6A levels in healthy skeletal muscles, which reduced skeletal muscle mass. On the contrary, the increase in m6A levels mediated by R-2HG alleviated denervation induced muscle atrophy. The m6A RNA methylation regulated skeletal muscle mass through ubiquitin–proteasome pathway.

**Conclusion:**

This study indicated that decrease in m6A RNA methylation was a new symptom of denervation-induced muscle atrophy, and confirmed that targeting m6A alleviated denervation-induced muscle atrophy.

**Supplementary Information:**

The online version contains supplementary material available at 10.1186/s12967-023-04694-3.

## Introduction

As a common clinical trauma, peripheral nerve injury can lead to severe skeletal muscle atrophy. Denervation-induced muscle atrophy progresses rapidly. Although it is possible to repair peripheral nerve defects, for long-distance nerve defects, due to the slow rate of nerve regeneration, irreversible atrophy often occurs before skeletal muscle is reinnervated, making it difficult to maintain skeletal muscle mass and function before reinnervation [1–3]. Therefore, it is crucial to find ways to alleviate skeletal muscle atrophy. Though, remarkable advance has been made in the investigation of underlying mechanisms of muscle atrophy for the last decades. The trigger factors and specific molecules in the muscle atrophy have not been fully elucidated.

As is well known, the essence of skeletal muscle atrophy is an imbalance between protein synthesis and protein degradation. During denervation-induced muscle atrophy, protein degradation pathways such as ubiquitin–proteasome system (UPS), autophagy-lysosome pathway (ALP), caspase pathway and calpain pathway are significantly activated [3–6]. Among them, Muscle RING Finger 1 (MuRF1) and Muscle Atrophy F-box (MAFbx, also known as Atrogin-1) are two key muscle specific E3 ubiquitin ligases that are significantly upregulated in various muscle atrophy models [7–10]. Transcriptome analysis found that in addition to the changes in protein expression related to protein synthesis and degradation pathways, there were also changes in many mRNA molecules during the process of denervation-induced muscle atrophy, involving epigenetics, angiogenesis, energy metabolism, oxidative stress, inflammation, polyamine metabolism, etc. [11–17]. Recent studies have found that many non-coding RNAs play an important role in denervation-induced muscle atrophy by regulating synthesis and degradation pathways [18–26]. In addition, alternative splicing (AS) is also involved in the denervation-induced muscle atrophy process, and multiple genes involved in degradation pathway have been reported to undergo splicing changes during the process of denervation-induced muscle atrophy [27]. From this, it can be seen that RNA metabolic disorders may be the trigger event for start transcriptional activity of proteolytic pathways in denervation-induced muscle atrophy.

It is worth noting that all RNA can be modified. Among the more than 150 known RNA modifications, N(6)-methylation of adenosine (m6A) is the most abundant RNA modification [28]. The m6A modification is a dynamic and reversible post-transcriptional modification, which mediated by 'writers' (methylase, adding methyl groups, methyltransferase-like3 (METTL3), METTL14, Wilms tumor 1associated protein (WTAP), and Vir Like m6A methyltransferase associated (VIRMA)) and ‘erasers’ [demethylase, deleting methyl groups, FAT mass and obesity-associated protein (FTO) and ALKB homologue5 protein (ALKBH5)]. In addition to writer and eraser, transcripts containing m6A are also recognized and managed (determining fate) by m6A binding proteins (Reader), including YTH domain-containing family protein 1–3 (YTHDF1/2/3) and YTH domain-containing protein 1–2 (YTHDC1/2) [29–31]. The m6A is involved in almost all metabolic processes of RNA from neogenesis to decay [32]. Therefore, it is easy to understand that m6A methylation plays a crucial role in many biological processes, such as development, aging, cell fate determination, and cancer occurrence [29]. In skeletal muscle, the deletion of muscle fiber specific conditional genes in the m6A writer METTL3 causes spontaneous muscle atrophy [33]. In skeletal muscle atrophy caused by denervation, the overloaded m6A eraser ALKBH5 activates the atrophy pathway through the HDAC4-FoxO3 axis, ultimately leading to muscle atrophy [34]. Although it is known that m6A changes driven by regulators can control skeletal muscle mass, it is not clear to what extent m6A changes are caused during the process of denervation-induced muscle atrophy and to what extent they are involved in the atrophy process.

In this study, we performed the m6A immunoprecipitation sequencing (MeRIP-seq) on skeletal muscles at multiple time points after denervation and described the dynamic changes in m6A modification. In addition, we manipulated the content of m6A in skeletal muscle using two drugs and found a negative correlation between m6A levels and skeletal muscle mass. Mechanistically, we found that m6A transcripts altered in the late stage of denervation-induced muscle atrophy are mainly associated with ubiquitin–proteasome pathway. These data provide a new perspective on the molecular mechanism of denervation-induced muscle atrophy, and also provide a basis for future anti-atrophy therapy targeting m6A.

## Materials and methods

### Animal feeding and management

The animal experiments involved in this project have been approved by the Animal Protection and Utilization Committee of Nantong University and the Jiangsu Provincial Animal Protection Ethics Committee (No. S20200312-003). All animals were kept in a specific pathogen free (SPF) level barrier system at the Experimental Animal Center of Nantong University. Animals were placed in an environment with optimal temperature (24 ± 2 ℃) and automatic control of light cycle, allowing for free access to food and water.

### Construction of the model of denervation-induced muscle atrophy

Thirty healthy adult male Sprague Dawley (SD) rats weighing 210 g ± 5 g were provided by the Experimental Animal Center of Nantong University. Rats were randomly divided into 10 groups (n = 3) to establish a sciatic nerve dissociation model. Preoperative weighing was performed and rats were anesthetized with 1% pentobarbital sodium at a dose of 0.3 ml/100 g body weight. Cut off 1.5–2 cm long nerves from the sciatic nerves of rats at the same location, then suture the skin with sutures soaked in 75% ethanol, and finally treat the surgical wound with iodophor. Starting from the time of nerve disconnection, the rats were euthanized using spinal dislocation method at 0 h, 0.25 h, 0.5 h, 1 h, 3 h, 6 h, 12 h, 24 h, 36 h, and 72 h, respectively. The tibialis anterior (TA) muscle was taken and weighed. The muscle tissue was soaked in liquid nitrogen and stored at − 80 °C for RNA extraction. The healthy adult male ICR mice weighing 30 g ± 2 g were provided by the Experimental Animal Center of Nantong University. All ICR mice were randomly divided into 5 groups (0–3 days), 6 groups (0–14 days) and 4 groups (drug treatment). The sciatic nerve dissociation models were established. Anesthetize mice with 1% pentobarbital sodium at a dose of 0.2 ml/10 g body weight. After preparing the corresponding samples, measure and calculate the muscle mass/body mass ratio (mg/g), the muscle wet weight ratio (denervated/contralateral mass ratio), and the muscle fiber cross-sectional area. Other methods refer to rats.

### Construction of MeIP library

MeRIP-Seq completed at OE Biotech (Shanghai, China). In short, the total RNA (400 μg, Qualified quality inspection) was purified twice using magnetic beads carrying oligo dT to capture mRNA with ploy (A) tail (polyadenylate). Fragmentation of Poly (A) RNA was performed in a buffer containing 10 mM ZnCl_2_. Fragmented RNA was divided into two parts, of which 10% was used as input control, and the rest RNA was subjected to m6A RNA immunoprecipitation (IP). RNA was first incubated with m6A polyclonal antibodies, followed by binding to pre-equilibrated m6A-Dynabeads. After multiple elutions, m6A-positive RNA was obtained using phenol–chloroform extraction and ethanol precipitation methods. Evaluate the quality of MeIP library using the BioAnalyzer 2100 system. Sequencing was performed on Illumina Hiseq to obtain a 150 bp double ended reading.

### Bioinformatics analysis

For raw data (raw reads) generated in high-throughput sequencing, firstly Trimmomatic software [35] was used to remove the connectors, then low-quality reads were filtered out, and finally high-quality clean reads were obtained. SortMeRNA software is used to remove ribosomal RNA [36]. Use the default parameters of HISAT2 software to compare clean reads to the reference genome of rats, while retaining the unique comparison reads for subsequent analysis. Using input samples as a control, MeTDiff software [37] was used for peak detection and differential analysis, with parameters of ‘FRAGMENT_LENGTH = 200, PEAK_CUTOFF_PVALUE = 0.01, PEAK_CUTOFF_FDR = 0.05’. The ChIPseeker software was used to annotate the detected peaks. MEME and DREME software were used to detect motifs in peak sequences, while Tomtom software was used to compare the obtained motif sequences with known motif databases and annotate them accordingly using known motifs. The GO database (www.geneontology.org) was used for GO analysis of differentially m6A genes. The rMATS software was used for variable splicing analysis.

### RNA extraction and Real time fluorescence quantitative PCR

According to instructions, total RNA was extracted from muscle tissue using TRIzol reagent (Vazyme, Nanjing, China), and the first strand cDNA was synthesized using a reverse transcription kit (Absin, Shanghai, China). Real time fluorescence quantitative PCR (qPCR) detection was performed using ChamQ Universal SYBR qPCR Master Mix (Vazyme, Nanjing, China) and BIO-RAD system (BIO-RAD-96CFX). Using 18 s as the internal reference, the relative mRNA expression level was detected using the 2^−∆∆Ct^ method. The mRNA primers were designed and synthesized by Shanghai Shenggong Biology Co., Ltd. The primer information is listed in Additional file 1: Table S1.

### Detecting m6A in muscle tissues

The EpiQuik m6A RNA Methylation Quantification Kit (GEPIGENTEK, Farmingdale, New York, USA) was used to analyze the total amount of m6A in the TA muscle of mice. Follow the manufacturer’s instructions, in short, mix the total RNA with a special binding solution to ensure that m6A modified RNA was captured by specific antibodies. After signal enhancement, the m6A RNA-antibody complex was determined by colorimetric quantification at 450 nm using an enzyme-linked immunosorbent assay (Bio-Tek, Vermont, USA).

### Immunohistochemistry

Mouse TA muscles were fixed with 4% paraformaldehyde (Beyotime, Shanghai, China) for 12 h, followed by sucrose gradient dehydration. Subsequently, the tissue was cut into 10 µm thick frozen sections using freezing microtome (CM3050S, Leica, Mannheim, Germany) and placed overnight in a 37 ℃ incubator. Slices were rinsed with PBS three times for 5 min each time. After adding an appropriate amount of blocking solution (Beyotime, Shanghai, China), the slices were sealed at room temperature for 1 h. They were incubated overnight with the first antibody anti-laminin antibody (1:1000, Abcam, Cambridge, UK) at 4 ℃, and then incubated with the second antibody Goat anti-mouse IgG H&L (1:500, Abcam, Cambridge, UK) at room temperature in dark for 2 h. Slices were sealed with sealing solution containing DAPI. An upright fluorescence microscope (Zeiss, Oberkochen, Germany) was used to capture fluorescence signals and obtain images. ImageJ software was used to measure the cross-sectional area of muscle fibers in various fields of view.

### Western blotting

Tissue samples were lysed with protein lysis buffer (Beyotime, Shanghai, China) added with phosphatase inhibitor mixture (Beyotime, Shanghai, China) and protease inhibitor mixture (Beyotime, Shanghai, China). The BCA detection kit (Beyotime, Shanghai, China) was used to determine protein concentration. Extracted samples (20 μg total proteins per lane) was separated by 10% sodium dodecyl sulfate polyacrylamide gel electrophoresis, then transferred to PVDF membrane (Millipore, Massachusetts, USA), sealed with Tris buffer saline containing 5% skimmed milk powder, and incubated with the primary antibody overnight at 4 ℃. The primary antibodies used in this study include anti-Myosin Heavy Chain/MHC antibody (1:1000, Abcam, Cambridge, UK), anti-Fbx32 antibody (1:1000, Abcam, Cambridge, UK), anti-beta Tubulin (1:1000, Abcam, Cambridge, UK), anti-MuRF1 antibody (1:1000, Abcam, Cambridge, UK), anti-ALKBH5 antibody (1:1000, Proteintech, UK), and anti-FTO antibody (1:1000, Proteintech, UK). The PVDF membrane was eluted multiple times in buffer saline containing 0.1% Tween-20, and incubated with goat anti-rabbit IgG H&L as secondary antibodies at room temperature for 2 h. Immunoblotting was visualized by High-sig ECL Western Blotting Substrate (Tanon, Shanghai, China). Use ImageJ software to analyze contrast and normalize it with reference bands. ImageJ software was used to analyze the grayscale values of immunoblotting bands and normalize the target band with the reference band.

### Drug treatment

3-deazidenosine (Daa) is an inhibitor of S-adenosylhomocysteine hydrolase, which has been proved to reduce m6A methylation in vivo [38, 39]. R-2-hydroxyglutarate (R-2HG) is a tumor metabolite, which can increase intracellular m6A levels by inhibiting the activity of FTO [40, 41]. 5% DMSO was used to dissolve the drug, with a final concentration of 0.04 mg/μl for Daa and 0.0012 mg/μl for R-2HG. TA muscle intramuscular multipoint injection were performed immediately after the sciatic nerve transection in drug treatment group mice. Each mouse was injected with 15 μl per day for a total of 7 days.

### Statistical analysis

All statistical analyses were performed using Prism 9 software (GraphPad, LaJolla, CA). All data are expressed as means ± standard deviation (SD) and analyzed using a one-way analysis of variance. Intergroup differences were detected using Tukey’s multiple comparisons test. A value of p < 0.05 was considered statistically significant.

## Results

### m6A features of pre-atrophic skeletal muscle

**Fig. 1:**
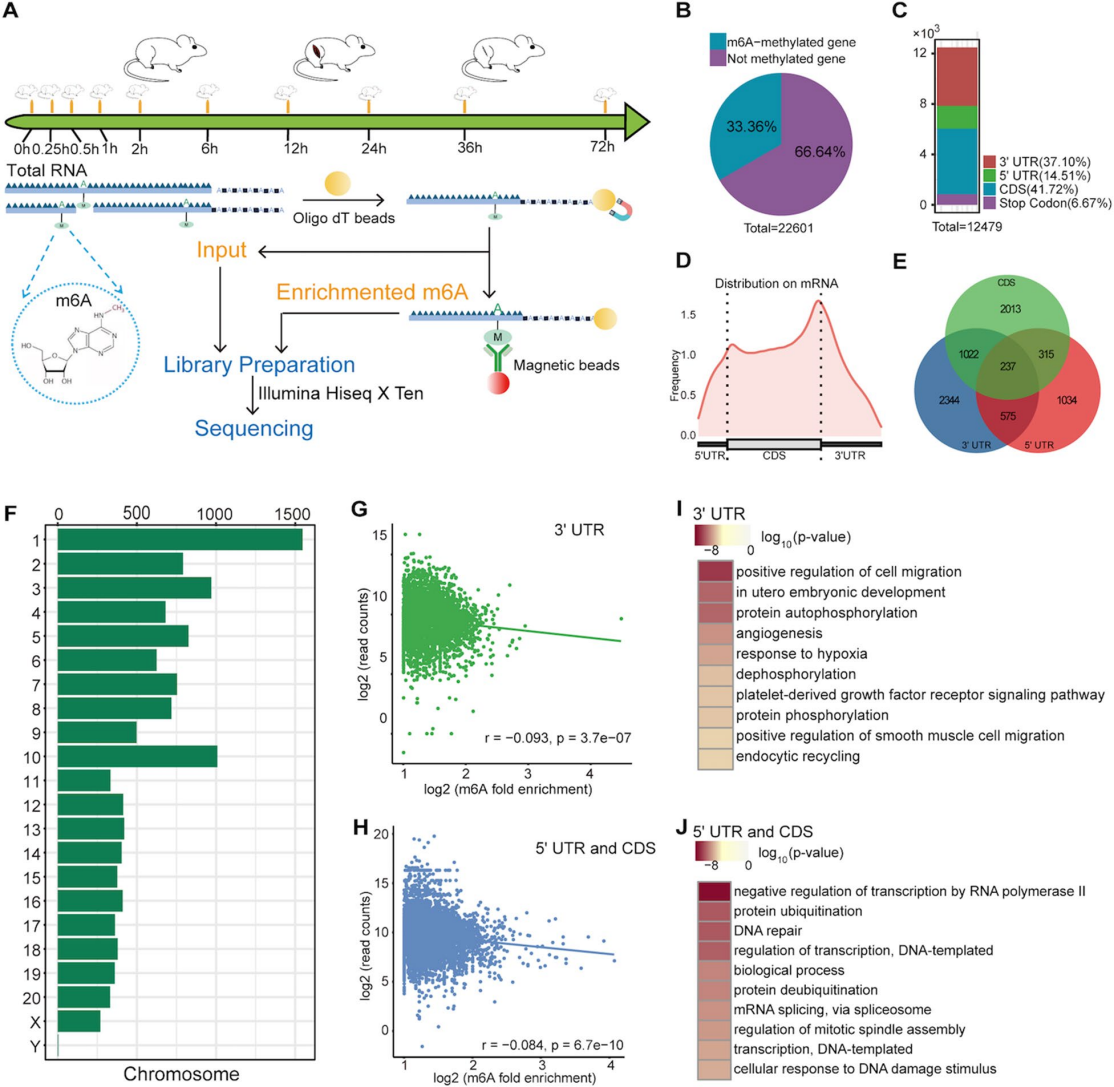
m6A features of pre-atrophic skeletal muscle. **A** A schematic diagram for the construction of the denervation-induced muscle atrophy model and MeRIP-seq library. **B** The proportion of genes with and without m6A methylated modification in pre-atrophic skeletal muscle. **C**, **D** The distribution of m6A peaks in various regions of mRNA transcripts. **E** The Venn diagram of m6A modified genes across transcription regions. **F** The distribution of m6A modification on different chromosomes. **G** The correlation analysis between the expression level of methylated transcripts and m6A in 3′ UTR. The x-axis represents the m6A enrichment multiple of log2 conversion, and the y-axis represents the read numbers of transcripts that undergos log2 conversion. H. Correlation analysis between the expression levels of transcripts containing methylation modifications and m6A in 5′ UTR and CDS. I. The functional analysis of 3′ UTR containing methylation modified genes. The darker the color, the higher the significance of this pathway. J. The functional analysis of 5′ UTR and CDS containing methylation modified genes

The m6A characteristics of skeletal muscles have been analyzed in human, mouse and pig, but the data from rats is lacking [42–47]. Therefore, we first focused on the characteristics of m6A modification in pre-atrophic rat skeletal muscles. Using m6A MeRIP, we constructed the m6A profile (IP) and conventional transcript profile (input) of rat skeletal muscles. The experiment setted up dense time gradients, including 0 h, 0.25 h, 0.5 h, 1 h, 3 h, 6 h, 12 h, 24 h, 36 h, 72 h after denervation, which would help to analyze the dynamic changes of m6A in detail (Fig. 1A).

Through strict screening criteria (fold enrichment ≥ 2, FDR ≤ 0.05), 12,479, 12,534, 12,173, 12,934, 11,773, 12,630, 10,136, 8953, 11,889, and 12,344 m6A peaks were defined at 0 h, 0.25 h, 0.5 h, 1 h, 3 h, 6 h, 12 h, 24 h, 36 h, and 72 h, respectively. They were associated with 7544, 7583, 7460, 7768, 7231, 7493, 6388, 5801, 7179, and 7458 transcripts (Additional file 1: Fig. S1A). For samples at different times, there was no significant difference in the median length (median and interquartile range) of m6A peak (Additional file 1: Fig. S1B). In skeletal muscle, the frequencies of GA-enriched motifs were higher among the fragments modified with m6A (Additional file 1: Fig. S1C). The distribution of detected m6A fragments and input fragments in the genes were different. The frequency of input was higher at the coding sequence (CDS), whereas the frequency of m6A fragment was higher at the junction of 5′ UTR and CDS (Additional file 1: Fig. S1D). The Principal Component Analysis (PCA) results showed that the data of m6A and input were gradually changed and could be clearly separated during skeletal muscle atrophy from early to mid to late stages (Additional file 1: Fig. S1E). Although the data at 12 h had a large degree of dispersion, according to general standards, expression differences could be distinguished based on expression levels, indicating that the data at 12 h did not affect differential gene recognition (Additional file 1: Fig. S1F).

The 7534 genes contained m6A modifications, accounting for approximately one-third of the total detected genes (Fig. 1B). The m6A peak was widely distributed in mRNA, with the higher distribution in CDS and 3′ UTR, and with the higher frequency at the junction of the two (Fig. 1C, D). Most genes were uniquely methylated by m6A, but 237 genes contained m6A methylation of 5′ UTR, 3′ UTR, and CDS simultaneously (Fig. 1E). Chr1, chr3, and chr10 emerged as the top three chromosomes with the highest frequency of modification, while chrY emerged as the least methylation modification (Fig. 1F).

It is known that m6A methylation can regulate the abundance of transcripts and also affect variable splicing [48, 49], both of which are closely related to denervation-induced muscle atrophy [11]. Here, we analyzed the correlation between m6A and gene expression, as well as variable splicing. Using rMATS, the numbers of variable splicing events were defined for the 0 h sample, including alternative 3′ splice site (A3SS, 3136), alternative 5′ splice site (A5SS, 1948), retained intron (RI, 29,354), skipped exon (SE, 26,171), and mutually exclusive exon (MXE, 283) (Additional file 1: Fig. S2A). The correlation between m6A and the two common variable splicing forms, SE and RI, was very low (r = 0.014, p = 0.25 and r = 0.0032, p = 0.6) (Additional file 1: Fig. S2B). According to distribution, m6A was divided into three types: 3′ UTR, CDS, and 5′ UTR, and correlation analysis of gene expression was conducted separately. There was a slight negative correlation between transcripts carrying m6A in 3′ UTR and expression levels (r = − 0.093, p = 3.7e^−7^), and a similar negative correlation (r = − 0.084, p = 6.7e^−10^) was also observed in transcripts carrying m6A in 5′ UTR and CDS (Fig. 1G, H). These data suggested that m6A methylation might negatively regulate gene expression but did not affect variable splicing. Gene Ontology (GO) analysis revealed the function of genes carrying m6A modification in healthy skeletal muscles. The genes modified by m6A in 3′ UTR were mainly related to biological processes such as positive regulation of cell migration, in utero embryonic development, protein autophosphorylation, and angiogenesis (Fig. 1I). The genes modified by m6A in 5′ UTR and CDS were mainly associated with negative regulation of transcription by RNA polymerase II, protein ubiquitination, DNA repair, regulation of transcription, DNA-templated, biological process, protein deubiquitination, and mRNA splicing via spliceosome, etc. (Fig. 1J).

### m6A methylation changes during denervation-induced muscle atrophy

**Fig. 2:**
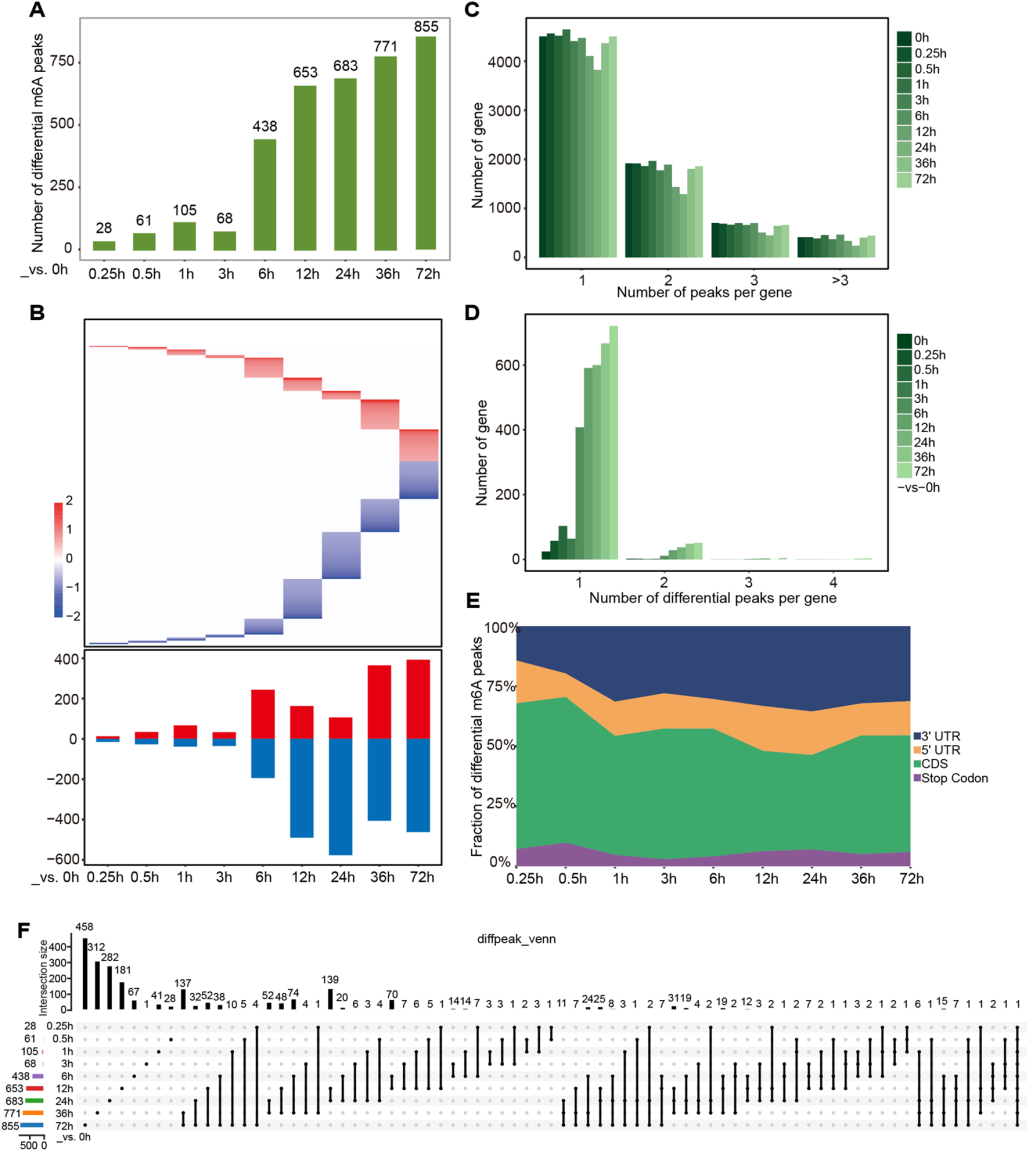
m6A methylation changes during denervation-induced muscle atrophy. **A** The number of differential m6A peaks at different time points during denervation-induced muscle atrophy. **B** The fold changes of differential m6A peaks at different time points. The above heat map displayed the distribution of the fold change, while the below column chart showed the number of m6A peaks up (red) and down (blue), respectively. **C** The proportion of host genes containing differential m6A peaks. **D** The proportion of genes containing varying numbers of differential m6A peaks at different time points. **E** The percentage of differential m6A distributed in different gene regions during denervation-induced muscle atrophy. **F** The Venn diagram of differential m6A peaks at different time points during denervation-induced muscle atrophy. (The intersections of m6A genes and convert the peak information into gene names to summarize the list in Additional file 2: File S1.)

In order to further investigate how m6A changes during denervation-induced muscle atrophy, we defined the differential m6A peaks based on strict criteria (fold change ≥ 2, FDR ≤ 0.05). The differential peaks were less in the initial stage of denervation, but significantly increased after 6 h. From 0.25 h to 72 h, 28, 61, 105, 68, 438, 653, 683, 771, and 855 differential m6A peaks were identified (Fig. 2A). At 6 h after denervation, the number and amplitude of down-methylated m6A peaks were higher than those of up-methylated m6A peaks in skeletal muscle, which was consistent with the results of decrease in m6A content at 72 h (Figs. 1F and 2B). About 50% of genes contained only one m6A peak, while the remaining 50% contained more than one m6A peak (Fig. 2C). For differential peaks, most of them only corresponded to one transcript, especially at 6 h (Fig. 2D). At 0.25 h and 0.5 h of atrophy, differential peaks were mainly distributed in the CDS region of mRNA. Over time, differential peaks gradually decreased in the CDS region, while differential peaks gradually increased in the 3′ UTR (Fig. 2E).

During skeletal muscle atrophy, there was only one host gene, *Myh9*, which underwent differential m6A modification at all time points. The 458 differential m6A peaks were 72 h specific and belonged to 402 host genes. The 137 differential m6A peaks were common at 36 h and 72 h, and they could be mapped to 95 genes (Fig. 2F, Additional file 2: File S1).

### m6A methylation regulates muscle gene expression after denervation

**Fig. 3:**
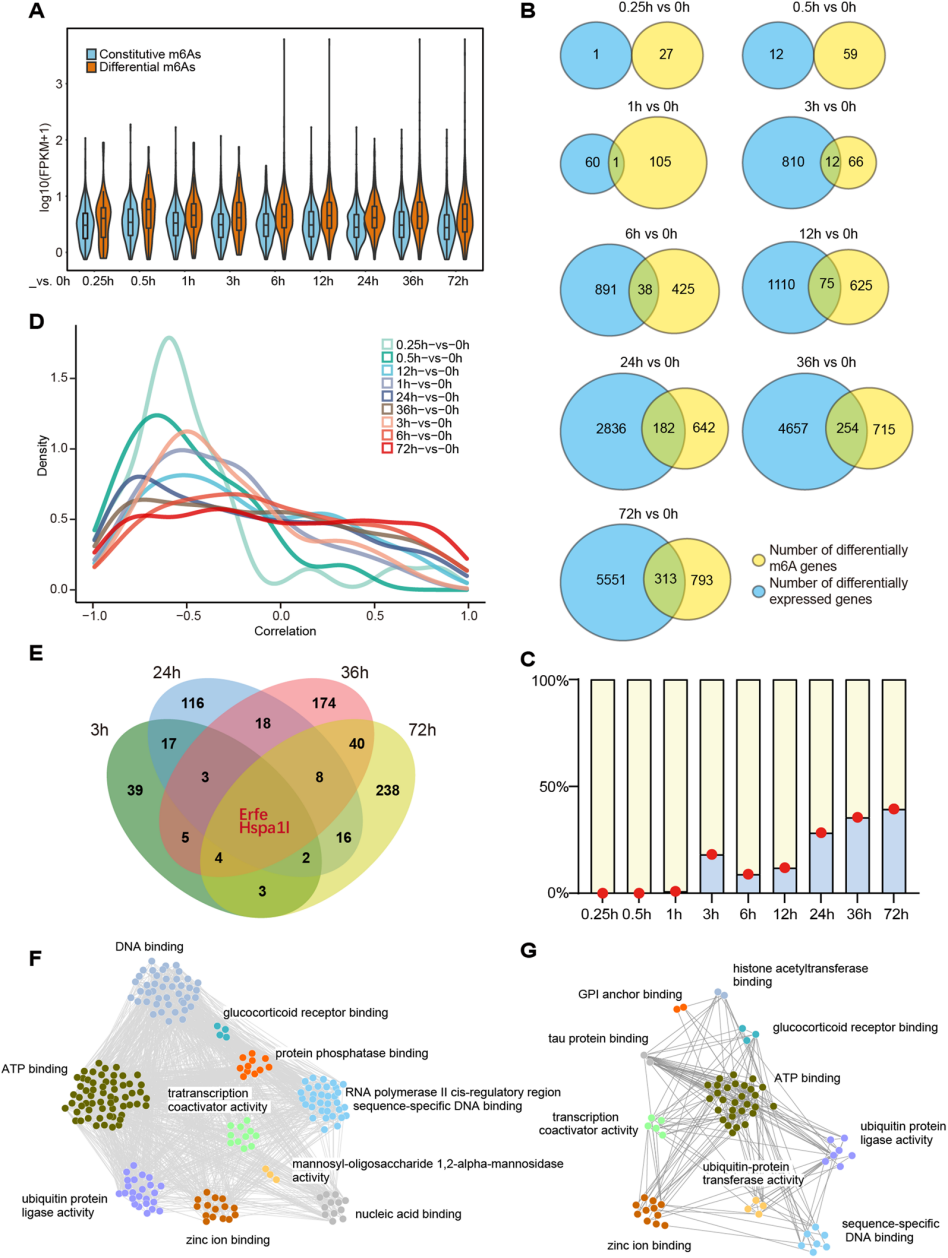
m6A methylation regulates muscle gene expression after denervation. **A** The expression levels of constitutive and differential m6A host genes. The violin diagram showed the maximum and minimum values, as well as the histogram of gene expression levels. The box plot in the middle represented the interquartile range. **B** The statistics of the numbers of differentially expressed genes (blue), differential m6A host genes (yellow), and overlapping genes (green) at different time points. **C** The proportion of overlapped genes in **B** among all differential m6A host genes (represented by red dots). **D** Correlation analysis between the differential m6A gene and its corresponding gene expression at 9 time points in **B**. The y-axis represented the density of the genes, while the x-axis centered around 0 represented negative correlation on the left and positive correlation on the right. E. For the four time points with the higher proportion in **C**, the overlapping genes, Erfe and Hspa1l, were differentially expressed genes regulated by m6A at each time point. **F** Functional analysis of differential m6A host genes at 72 h of denervation. The biological processes of TOP10 were displayed, and the lines indicated the interactions between genes. **G** Functional analysis of m6A methylated differentially expressed genes at 72 h of denervation. The biological processes of TOP10 were displayed, and the genes were analyzed by PPI. The lines indicated the interactions between genes

We examined the relationship between differential m6A modifications and gene expression during denervation-induced muscle atrophy. The results showed that the expression levels of differential m6A methylated genes were generally higher than those of constitutive m6A methylated genes (Fig. 3A). To further analyze the relationship between m6A and gene expression, we counted differentially expressed genes and differential m6A genes at various time points and extracted their intersection genes (Fig. 3B). At early time points (0.25 h, 0.5 h, 1 h), the proportion of intersection genes in the differential m6A genes approached 0, indicating that m6A changes in the early stage of denervation did not affect gene expression (Fig. 3B, C). At later time points (24 h, 36 h, 72 h), this proportion of intersection increased to about 40%, indicating that about 40% of m6A modified genes were differentially expressed in the late stage of denervation (Fig. 3B, C). The correlation analysis results showed that there was a negative correlation between differential m6A gene expression and m6A modification at different time points (Fig. 3D). There were two genes, *Erfe* and *Hspa1l*, which exhibited both differential m6A modification and differential expression at various time points in the late stage of denervation (Fig. 3E). *Erfe* (also known as Ctrp15) encodes a skeletal muscle-derived myonectin, which can improve acute myocardial injury by regulating energy metabolism [50, 51]. *Hspa1l* encodes a heat shock protein involved in autophagy regulation [52, 53]. Although both them are related to skeletal muscle physiology, they have not been reported in denervation-induced muscle atrophy.

By clustering host genes labeled with differential m6A into biological pathways, we observed the function of the differential m6A genes after 72 h of denervation. The 1106 differential m6A genes were mainly related to biological processes such as zinc ion binding, ubiquitin protein ligase activity, transcription coactivator activity, ATP binding, RNA polymerase II cis-regulatory region sequence-specific DNA binding, protein phosphatase binding, nucleic acid binding, glucocorticoid receptor binding, DNA binding, and mannosyl-oligosaccharide 1,2-alpha-mannosidase activity (Fig. 3F). The 313 differential m6A intersection genes were mainly related to biological processes such as zinc ion binding, tau protein binding, histone acetyltransferase binding, sequence-specific DNA binding, GPI anchor binding, glucocorticoid receptor binding, transcription coactivator activity, ATP binding, and ubiquitin protein ligase activity (Fig. 3G). It was worth noting that common pathways included zinc ion binding, ubiquitin protein ligase activity, ATP binding, sequence-specific DNA binding, and transcription activator activity, suggesting that m6A might affect the related gene expressions of ubiquitin–proteasome pathway and transcriptional activation during denervation-induced muscle atrophy.

### Increased m6A methylation level alleviates denervation-induced muscle atrophy

**Fig. 4:**
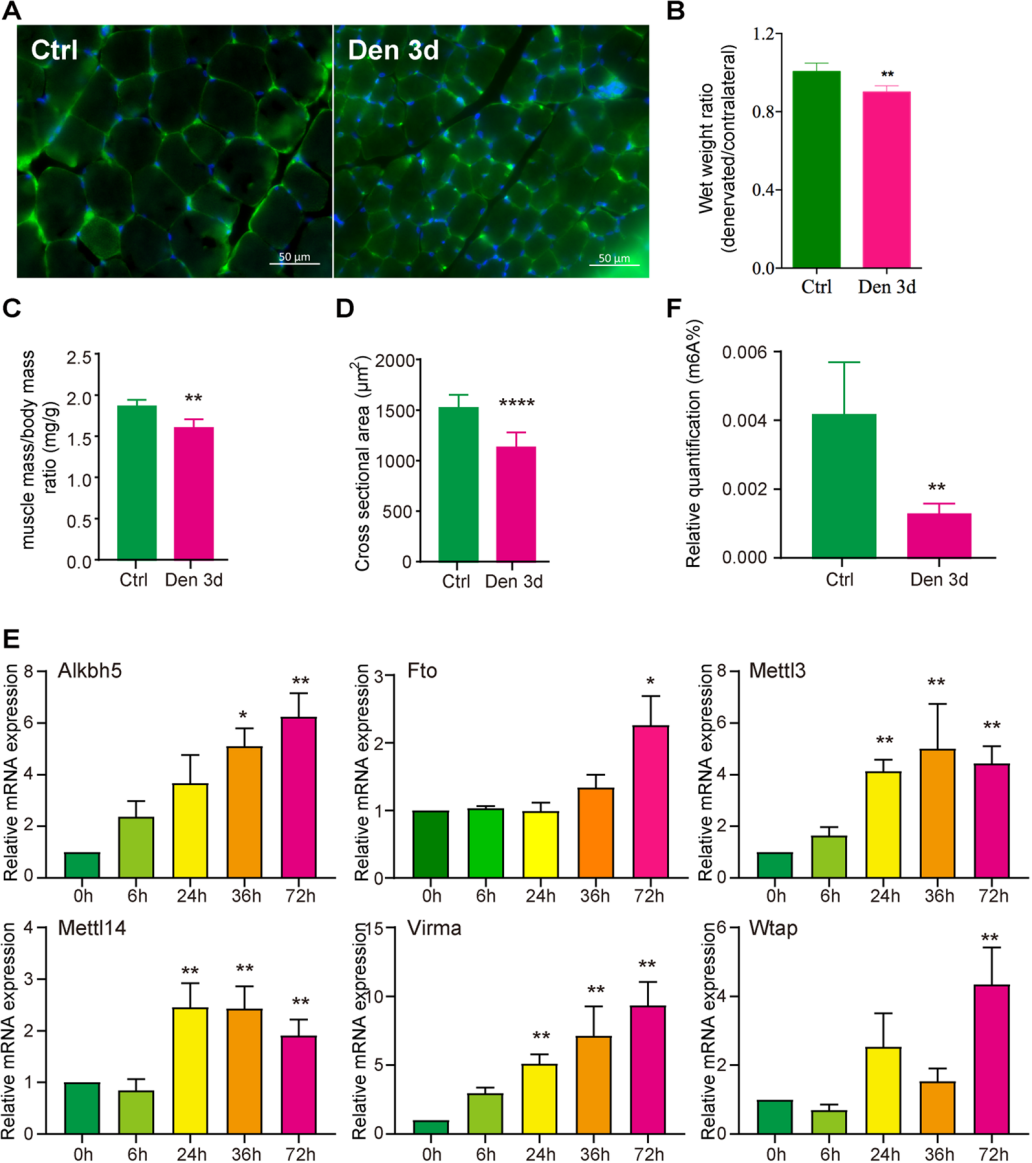
Changes in m6A regulators expression during denervation-induced muscle atrophy. **A** Immunofluorescence staining of skeletal muscles after denervation for 0 h (Ctrl) and 72 h (Den 3d), Bar = 50 μm, blue: DAPI, green: laminin. **B** Statistical results of wet weight ratio after denervation for 0 h (Ctrl) and 72 h (Den 3d). **C** Statistical results of the muscle mass/body mass ratio after 0 h and 72 h of denervation, n = 5. **D** Statistical results of cross-sectional area of skeletal muscle fibers after 0 h and 72 h of denervation, n = 5. **E** The qPCR results of several common m6A regulators, Alkbh5, Fto, Mettl3, Mettl14, Virma, and Wtap, during the denervation-induced muscle atrophy, n = 3. **F** The statistical results of m6A levels in skeletal muscle after 0 h and 72 h of denervation, n = 4. The p values were calculated through unpaired t-test, *p < 0.05, **p < 0.01, ****p < 0.0001. Unmarked columns have no statistical significance

To verify the relationship between m6A methylation levels and denervation-induced muscle atrophy, we established the sciatic nerve transection mouse model and dissected the tibialis anterior muscles. Morphological analysis of skeletal muscle showed significant decreases in the muscle mass/body mass ratio and muscle fiber cross sectional area after 72 h of denervation (Den 3d), compared to 0 h of denervation (Ctrl) (Fig. 4A–D). Then, we used RT-qPCR to analyze the expression changes of m6A regulators at 0 h, 6 h, 24 h, 36 h and 72 h after denervation (Fig. 4E). One of the erasers of m6A, *Fto*, showed significant increased expression, and the expression of another eraser, *Alkbh5*, gradually increased and was upregulated about sixfold at 72 h. The expressions of m6A writers, *Mettl3* and *Mettl14*, did not show significant changes at 6 h, but were upregulated at 24 h, 36 h and 72 h, respectively. The expression of m6A writers, *Virma*, was gradually increased, and upregulated about ninefold at 72 h. The expression changes of m6A writers, *Wtap*, exhibited fluctuations during atrophy. These results indicated that the changes of m6A modification were complex during denervation-induced muscle atrophy. The m6A RNA Methylation Quantification Kit was used to detect the content of m6A in skeletal muscle. Compared with the control group, the proportion of m6A decreased by about 70% at 72 h after denervation, indicating that the demethylation of mRNA was dominant in the late stage of denervation-induced muscle atrophy (Fig. 4F). Based on the qPCR results, we speculated that reduction of m6A might be mediated by Alkbh5 in the late stage of atrophy.

Then, we further examined the expression changes of demethylase during denervation-induced muscle atrophy at six time points: 0 day, 1 day, 3 day, 5 day, 7 day, and 14 day after denervation. The results showed that there were significant decreases in muscle mass/body mass ratio and wet weight ratio after 3 days of denervation (Additional file 1: Fig. S3A, B). The expression levels of Alkbh5 and Fto showed upward trends with prolonged denervation, with both significantly higher levels at 7 days of denervation (Additional file 1: Fig. S3C). These indicated that the expression of Alkbh5 and Fto were negatively correlated with the degree of skeletal muscle atrophy.

**Fig. 5:**
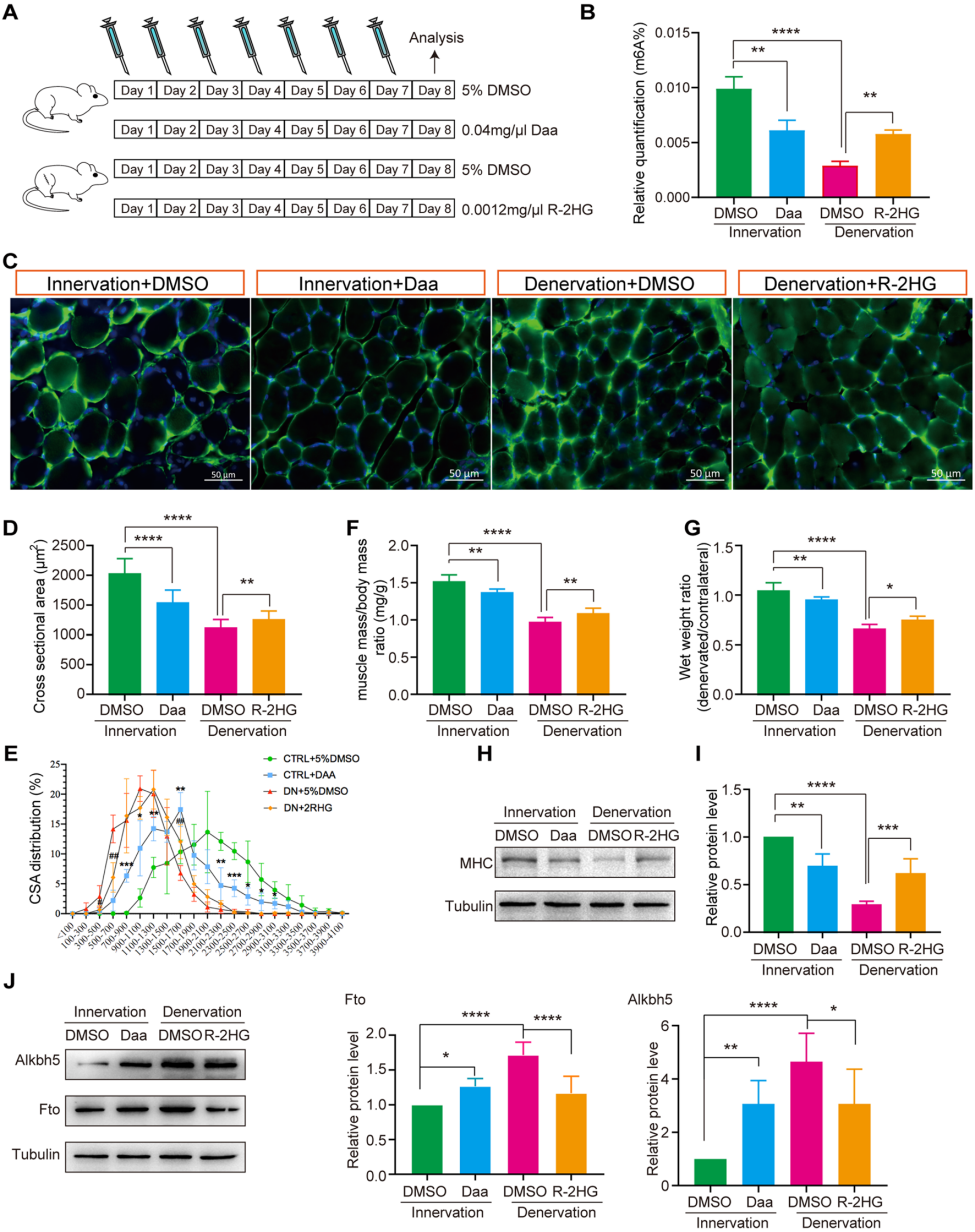
Increased m6A methylation level alleviates denervated muscle atrophy. **A** Schematic diagram of drug injection. **B** The levels of m6A in skeletal muscle after drug injection, *p*-value calculated through unpaired t-test, n = 4, **p < 0.01. **C** Immunofluorescence staining results of skeletal muscles after drug injection, Bar = 50 μm, blue: DAPI, green: laminin. **D** Statistical results of cross-sectional area of skeletal muscle fibers. **E** The distribution of muscle fiber cross sectional area after different drug treatments, *p*-value calculated through unpaired t-test, n = 4, *p < 0.05, **p < 0.01, ***p < 0.001, ****p < 0.0001 vs Inn + DMSO group; ^#^*p* < 0.05, ^##^*p* < 0.01, ^###^*p* < 0.001 vs Den + DMSO group. **F** Statistical results of the muscle mass/body mass ratio after drug treatment. **G** Statistical results of skeletal muscle wet weight ratio after drug treatment. **H** Western blot results of MHC expression after drug treatment. **I** Statistical results of MHC protein level. **J** Western blot and statistical results of Alkbh5 and Fto levels after drug treatment. *p*-value calculated through unpaired t-test, n = 3, *p < 0.05, **p < 0.01, ***p < 0.001, ****p < 0.0001

To further confirm whether m6A methylation played a role in denervation-induced muscle atrophy, we used two drugs to alter the content of m6A in skeletal muscle. We injected 0.04 mg/μl Daa into the target muscle of normal mice (innervation group) once a day for 7 consecutive days. At the same time, we constructed denervated animal model (denervation group), and the target muscle were injected with 0.0012 mg/μl R-2HG once a day for 7 consecutive days (Fig. 5A). Two experiments were conducted by injecting equal volumes of DMSO as control, and all mice were euthanized on the eighth day for subsequent experiments. We first tested the effectiveness of two drugs. The results showed that compared to the control group, Daa reduced the content of m6A by about 40% in normal skeletal muscles, while R-2HG increased the content of m6A by about onefold in denervated skeletal muscles (Fig. 5B). Morphological and statistical results showed that Daa significantly reduced the muscle fiber cross sectional area, the muscle mass/body mass ratio, and wet weight ratio of normal mouse muscles (Fig. 5C–E). The effect of R-2HG was opposite to that of Daa, as it significantly increased the muscle fiber cross sectional area, the muscle mass/body mass ratio, and wet weight ratio of denervated mouse muscles (Fig. 5C–E). In addition, we detected the expression of myosin heavy chain (MHC). The results showed that denervation resulted in a significant downregulation of MHC in skeletal muscles. Daa significantly inhibited the expression of MHC in normal skeletal muscles, while R-2HG significantly increased the expression of MHC in denervated skeletal muscles, consistent with morphological results (Fig. 5H, I). At the same time, we detected the expression changes of Alkbh5 and Fto. The results showed that the expression levels of both them were positively correlated with the degree of muscle atrophy, and this result was consistent with the relative m6A levels (Fig. 5J). Our results suggested that whether skeletal muscle atrophy was controlled by the level of m6A. On the other hand, R-2HG might be a potential drug for treating denervation-induced muscle atrophy.

### m6A methylation regulates ubiquitin–proteasome system

**Fig. 6:**
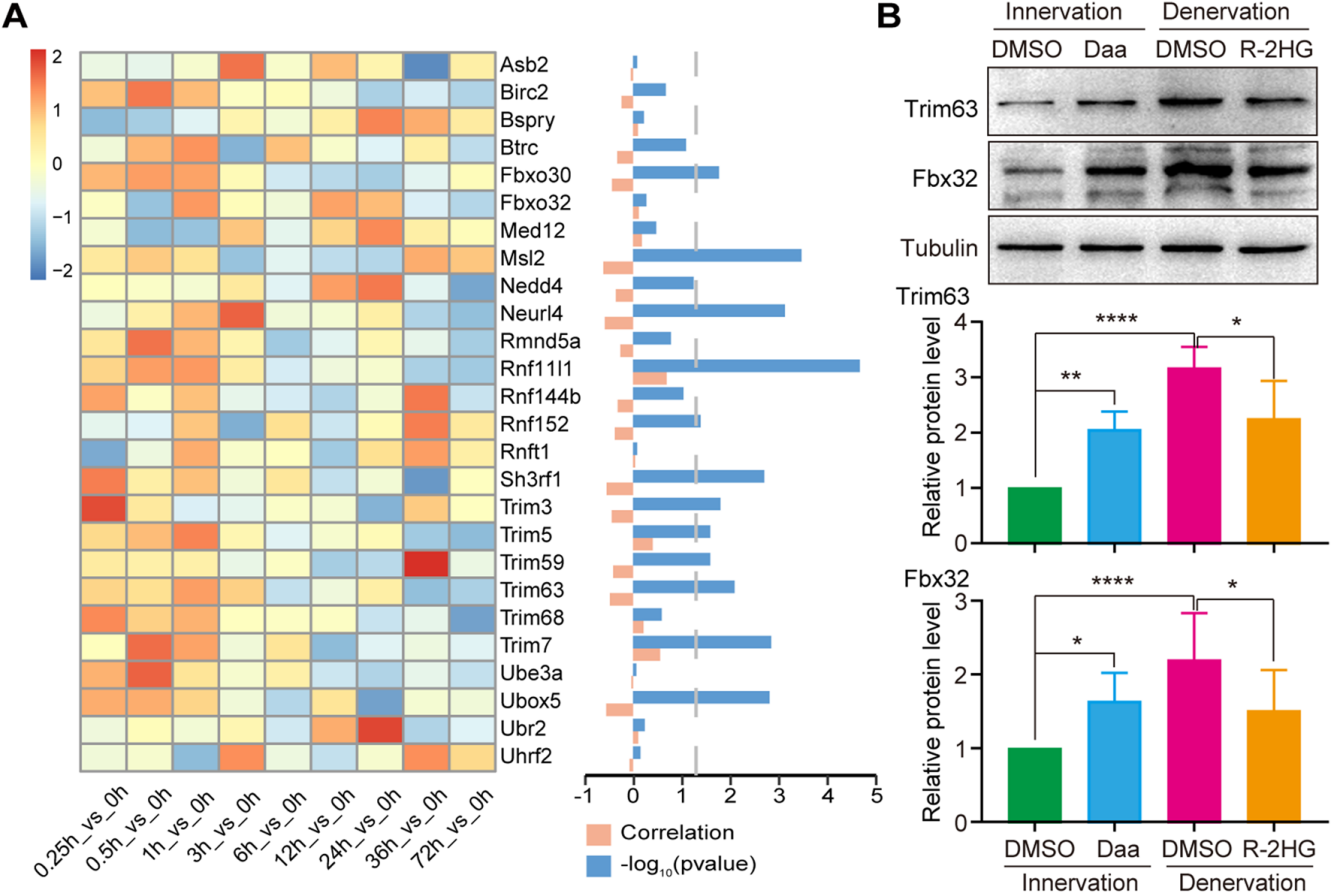
m6A methylation regulates ubiquitin proteasome system. **A** Expression analysis of ubiquitin proteasome related genes. The heat map on the left showed the expression changes of the ubiquitin proteasome related genes with differential m6A methylation during muscle atrophy. The orange column in the right figure represented the correlation between gene m6A modification and gene expression, while the blue column represented the *p*-value corresponding to the correlation. **B** The effects of Daa and R-2HG on the levels of two E3 ubiquitin ligases (Trim63 and Fbx32). Tubulin was used as a loading control, *p*-value calculated through unpaired t-test, n = 5, *p < 0.05, **p < 0.01, ****p < 0.0001

We next explored the mechanism of m6A regulation in denervation-induced muscle atrophy. The above results suggested that the function of differential m6A methylated genes was mainly related to the ubiquitin–proteasome system (Fig. 3F, G). We analyzed the correlation between the expression of m6A methylated genes of and m6A modification during denervation-induced muscle atrophy. The results showed a negative correlation between m6A modification and expression of most ubiquitin–proteasome related genes (Fig. 6A). Based on the discovery of a decrease in total m6A content during denervation-induced muscle atrophy, we speculated that the decreased m6A methylation might activate the ubiquitin–proteasome system, ultimately leading to the atrophy phenotype.

To validate this, we detected the effect of m6A changes on expressions of two key E3 ubiquitin ligases. The results showed that Daa significantly increased the expressions of *Trim63* and *Fbx32* in normal skeletal muscles, while R-2HG significantly downregulated the expression of them in denervated skeletal muscles (Fig. 6B). The expressions of *Trim63* and *Fbx32* were significantly negatively correlated with the content of m6A in skeletal muscles, and also matched the phenotype of muscle atrophy. Our results indicated that m6A modification regulated denervation-induced muscle atrophy through ubiquitin–proteasome system.

## Discussion

During denervation-induced muscle atrophy, the changes of transcriptome were dynamically regulated and highly coordinated by multiple mechanisms. Recently, m6A methylation has been confirmed to be involved in the development and management of skeletal muscle in multiple species [33, 34, 54–57]. In this study, we described the dynamic landscape of the m6A transcriptome in denervation-induced muscle atrophy. The results showed that m6A levels showed a decreasing trend during denervation-induced muscle atrophy. Moreover, m6A methylation was negatively correlated with gene expression, and a large number of upregulated ubiquitin–proteasome pathway related genes carried m6A modifications. More importantly, we confirmed that increasing m6A methylation was a potential treatment strategy for denervation-induced muscle atrophy.

This study designed dense time points for sequencing, which fully described the dynamics of m6A during denervation-induced muscle atrophy as much as possible. Furthermore, we focused on the m6A characteristics of pre-atrophic skeletal muscles, identified m6A modified genes, and analyzed their functions. Overall, these high-quality data provided valuable resources for further exploration of the role of m6A methylation in skeletal muscle atrophy.

The main effect of m6A on mRNA is to affect its stability [58, 59]. However, the relationship between m6A and gene expression has not been well explored during denervation-induced muscle atrophy. Our results showed that the relationship between m6A and gene expression was negatively correlated in both healthy and atrophic skeletal muscles, further confirming the role of m6A in degrading mRNA. The correlation in skeletal muscle is different from that in myocardium, indicating that m6A plays different roles in different tissues [60]. Nearly half of the differentially expressed m6A methylated genes undergone expression changes in the late stages of denervation skeletal muscle atrophy. They were negatively correlated with m6A abundance and can be significantly enriched in pathways such as the ubiquitin proteasome, and thus m6A also acts as a brake to avoid hyper activation of the proteolytic pathways. However, differential m6A methylation genes did not overlap with differentially expressed genes at early stages of denervation suggesting a differential role for m6A at different stages of denervation. In the late stage, m6A mediated mRNA degradation is more involved, and m6A modified in 3′ UTR is more involved in mRNA decay [61, 62]. Our results showed that 3′ UTR contained an increasing number of m6A peaks as muscle atrophy progresses, which was consistent with above.

Research has shown that the level of m6A controls skeletal muscle size. Increased expression of m6A writer Mettl3 using genetic approaches, and concomitantly m6A overload, caused hypertrophy of skeletal muscle and conversely caused spontaneous muscle atrophy [33]. The upregulation of m6A eraser ALKBH5 expression and reduction of m6A levels are associated with skeletal muscle atrophy caused by denervation [34]. Consistent with report, our study provided three direct evidences to confirm a positive correlation between the total level of m6A in skeletal muscle and skeletal muscle mass. Firstly, the level of m6A decreased after 3 days of denervation in skeletal muscle. Secondly, R-2HG increased m6A levels to rescue denervation-induced muscle atrophy. Thirdly, Daa reduced m6A levels in healthy skeletal muscles, leading to occurrence of skeletal muscle atrophy. Our results once again confirmed the positive correlation between m6A levels and skeletal muscle mass. Moreover, these also suggested that m6A decrease might be a common feature during all types of skeletal muscle atrophy.

Skeletal muscle contains multiple cell types, and it is not yet clear which cell type of m6A changes affect muscle atrophy. Muscle stem cells (MuSC) are crucial for skeletal muscle homeostasis and muscle regeneration after injury. Numerous studies have focused on the effects of m6A on the fate of muscle stem cells and muscle regeneration. METTL3 has high expression levels in proliferation and differentiation stages of MuSC, indicating that METTL3 induced high levels of m6A are involved in myogenesis [63]. Gheller et al. demonstrated that overall m6A levels increased in the early stage of skeletal muscle regeneration in vivo, and knocking down m6A methyltransferase METTL3 levels downregulated overall m6A levels, leading to premature differentiation of C2C12 myoblasts [42]. MuSC specific knockout of METTL3 significantly inhibited MuSC proliferation and blocked muscle regeneration after injury in mice, but the knockin of METTL3 promoted them [64]. Contrary to METTL3, the silencing of FTO inhibited the differentiation of MuSC, leading to impaired skeletal muscle development [65]. In addition, m6A reader Ythdc1 has been reported to be crucial for MuSC proliferation, but it is unclear whether this is mediated by m6A changes [56, 66]. In the present study, however, it is not clear which cell type contributes to the m6A changes in the tibialis anterior muscle tissue; it could be MuSC, myofibers or others. This interesting issue deserves to be explored in the future.

UPS activation is a direct characteristic of skeletal muscle atrophy initiation. Previous studies had mostly focused on the upstream signaling mediated UPS transcriptional activation, such as ROS, IL6, etc., neglecting the activation mechanism of UPS itself [14, 67, 68]. Our previous research found that the variable splicing changes of UPS related genes might lead to UPS activation during denervation-induced muscle atrophy [11, 27]. In this study, we proposed a new mechanism for autonomous activation of UPS, which involved the reduction of m6A modifications in UPS mRNAs during denervation-induced muscle atrophy (especially in the late stage of atrophy), leading to the activation of UPS. It is worth noting that the common genes with differential m6A modification and differential expression are also involved in transcriptional activation related pathways. Transcription factors play crucial roles in the process of neurogenic muscle atrophy, with the Forkhead box O (FoxO) family being extensively studied [69, 70]. FoxO3 can drive the expression of most atrogenes, including UPS and ALP [71–73]. Our results suggested that the expressions of many transcriptional activation related genes were regulated by m6A, which might indirectly activate UPS. Although there is still a lack of experimental evidence, this may still be one of the important regulatory mechanisms of m6A on denervation-induced muscle atrophy.

The regeneration and decay of m6A are the results of expression changes of writers and erasers. During denervation-induced muscle atrophy, both writers and erasers showed upward expression trends, indicating that the m6A changes were quite complex over time and did not change in a single direction. The expression of m6A eraser FTO showed increased change, and the expression of ALKBH5 showed a gradual upward trend, which were consistent with the study by LIU et al*.* [34]. Based on the overall decrease in m6A, it seemed that the upregulation of ALKBH5 expression was driving the m6A decrease during denervation-induced muscle atrophy. LIU et al. also confirmed that ALKBH5 deletion alleviated denervation-induced muscle atrophy. Both FTO and ALKBH5 have been reported to affect the expression of multiple atrophy genes or the activation of atrophy pathways. ALKBH5 drives skeletal muscle mass reduction after denervation through direct demethylation of HDAC4 and activation of the FoxO3 pathway [34]. FTO indirectly increases PGC-1α expression through demethylation of intermediate molecules, leading to fiber conversion in diet-induced skeletal muscle fiber remodeling [74]. In addition, FTO and ALKBH5 cooperatedly activate the downstream FOXO signaling pathway, which in turn affects cell proliferation [75]. Interestingly, the target of R-2HG is FTO, which does not affect the activity of ALKBH5, and still alleviates denervation-induced muscle atrophy. Although ALKBH5 and FTO both recognize RNA containing m6A, they exhibit key differences in substrate preference, intracellular localization, and the products [76–78]. Therefore, we proposed that the protective effect of m6A on denervation-induced muscle atrophy was not enzyme dependent, but depended on its overall m6A level.

The m6A exhibits therapeutic potential in many diseases [32]. R-2HG is an FTO targeted inhibitor that has been shown to significantly increase m6A levels and inhibit the development of glioma and leukemia [40, 41]. This study demonstrated the potential application of R-2HG (or m6A targeted therapy) during denervation-induced muscle atrophy, in addition to anti-tumor therapy. In addition to R-2HG, other FTO inhibitors (or m6A activators), such as Meclofenamic acid, N-(5-Chloro-2,4-dihydroxyphenyl)-1-phenylcyclobutanecarboxamide, as well as some small molecules, also have potential in anti-muscular atrophy effect [17, 79, 80], and are worth verifying in the future.

In summary, our research established a unique data resource for the study of denervation-induced muscle atrophy. Through data analysis combined with experimental verification, we not only analyzed the dynamic changes of m6A but also confirmed its role, which deepened our understanding of the molecular mechanism in denervation-induced muscle atrophy. Moreover, our data also emphasized that R-2HG was a potential treatment drug during denervation-induced muscle atrophy.

### Supplementary Information

**Additional file 1: Figure S1.** General description of the MeRIP-seq library. The numbers of m6A peaks and host genes detected at various time points of denervation. **B** Violin diagram of the m6A peak length. **C** The motif characteristics of m6A peak at various time points. **D** The distribution on mRNA of sequence fragments in IP and Input libraries. **E** PCA results for IP and Input data. **F** The heat map showed the differentially expressed genes at 0 h and 12 h of denervation. **Figure S2.** Correlation analysis of m6A methylation and variable splicing. **A** The number of different variable splicing patterns. **B** Correlation analysis between SE or RI and m6A methylation. A3SS: alternative 3′ splice site, A5SS: alternative 5′ splice site, MXE: mutually exclusive exon, RI: retained intron, SE: skipped exon. **Figure S3.** Changes in demethylase expression during denervated muscle atrophy. **A** Muscle mass/body mass ratio in different time points. **B** Wet weight ratio in different time points. **C** Expression of Alkbh5 and Fto in target muscles. Data were expressed as mean ± SD, n = 5, *P < 0.05 versus Ctrl group; **P < 0.01 versus Ctrl group. **Table S1.** List of PCR primer sequences.

**Additional file 2.** m6A methylation differential genes and their overlapping genes at different time points during denervation.

## Data Availability

The data that support the findings of this study are available from the corresponding author upon reasonable request.

## Abbreviations

m6A: N6-methyladenosine
UPS: Ubiquitin–proteasome system
ALP: Autophagy-lysosome pathway
METTL3: Methyltransferase-like3
WTAP: Wilms tumor 1associated protein
VIRMA: Vir Like m6A methyltransferase associated
FTO: FAT mass and obesity-associated protein
ALKBH5: ALKB homologue5 protein
AS: Alternative splicing
TA: Tibialis anterior
FoxO: Forkhead box O
Daa: 3-Deazidenosine
R-2HG: R-2-hydroxyglutarate
MuSC: Muscle stem cells
MuRF1: Muscle RING Finger 1
MAFbx: Muscle Atrophy F-box
AS: Alternative splicing
MHC: Myosin heavy chain
