# Supplementary material for: Altered m6A RNA methylation governs denervation-induced muscle atrophy by regulating ubiquitin proteasome pathway
Source: J Transl Med. 2023 Nov 23;21:845. doi: 10.1186/s12967-023-04694-3 (PMC10668433; doi:10.1186/s12967-023-04694-3)
Supplement: Supplementary file 1 — Additional file 1: Figure S1. General description of the MeRIP-seq library. The numbers of m6A peaks and host genes detected at various time points of denervation. B Violin diagram of the m6A peak length. C The motif characteristics of m6A peak at various time points. D The distribution on mRNA of sequence fragments in IP and Input libraries. E PCA results for IP and Input data. F The heat map showed the differentially expressed genes at 0 h and 12 h of denervation. Figure S2. Correlation analysis of m6A methylation and variable splicing. A The number of different variable splicing patterns. B Correlation analysis between SE or RI and m6A methylation. A3SS: alternative 3′ splice site, A5SS: alternative 5′ splice site, MXE: mutually exclusive exon, RI: retained intron, SE: skipped exon. Figure S3. Changes in demethylase expression during denervated muscle atrophy. A Muscle mass/body mass ratio in different time points. B Wet weight ratio in different time points. C Expression of Alkbh5 and Fto in target muscles. Data were expressed as mean ± SD, n = 5, *P < 0.05 versus Ctrl group; **P < 0.01 versus Ctrl group. Table S1. List of PCR primer sequences. [file 12967_2023_4694_MOESM1_ESM.docx]

**Supplementary Materials**

Supplemental Information includes figures and one tables.

Sup-Figure 1. General description of the MeRIP-seq library

Sup-Figure 2. Correlation analysis of m6A methylation and variable splicing

Sup-Figure 3. Changes in demethylase expression during denervated muscle atrophy

Sup-Table 1. List of PCR primer sequences.


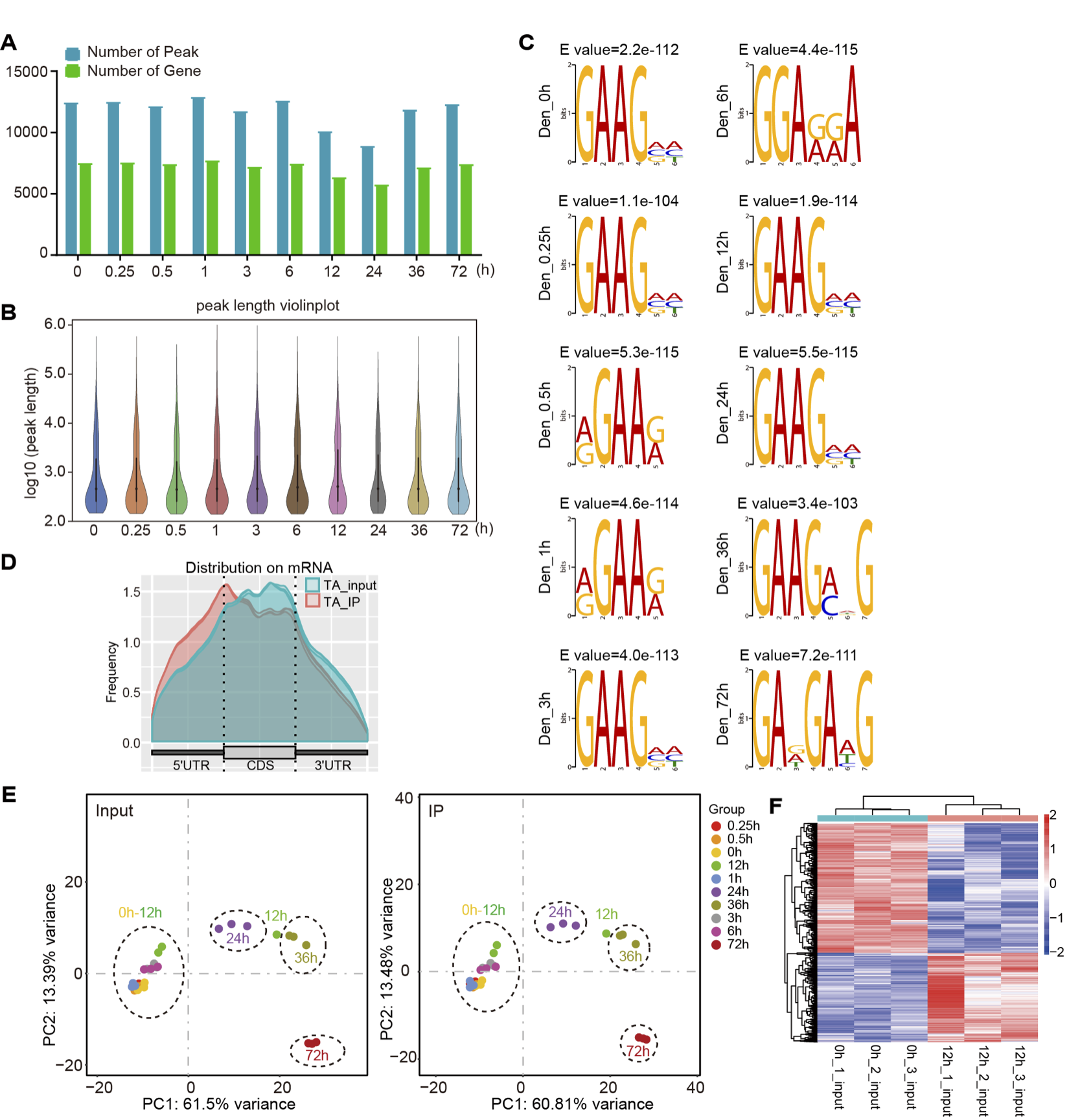


**Sup-****Figure 1. General description of the MeRIP-seq library**

The numbers of m6A peaks and host genes detected at various time points of denervation. B. Violin diagram of the m6A peak length. C. The motif characteristics of m6A peak at various time points. D. The distribution on mRNA of sequence fragments in IP and Input libraries. E. PCA results for IP and Input data. F. The heat map showed the differentially expressed genes at 0h and 12h of denervation.


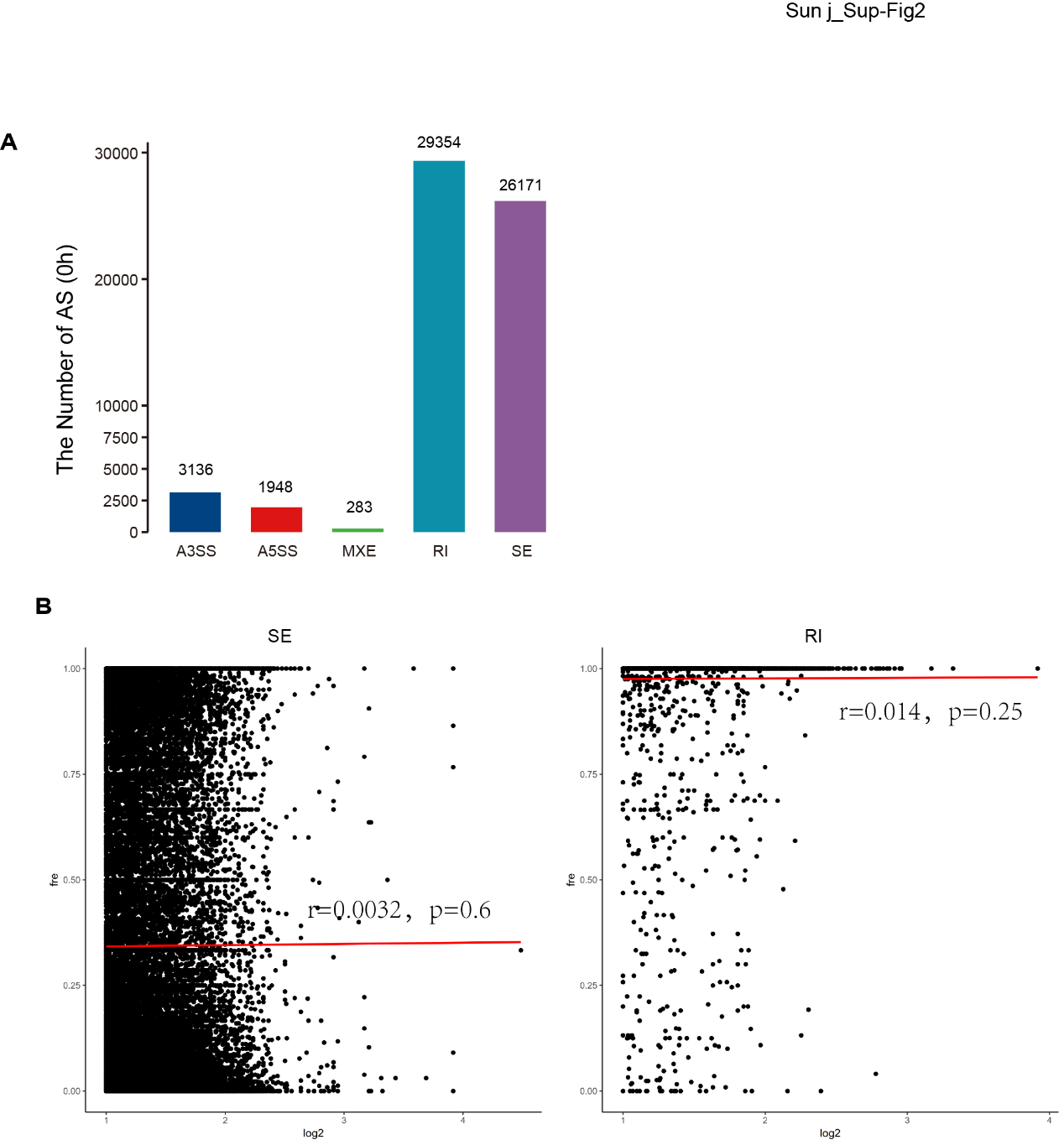
**Sup-Figure 2. Correlation analysis of m6A methylation and variable splicing**

A. The number of different variable splicing patterns. B. Correlation analysis between SE or RI and m6A methylation. A3SS: alternative 3′ splice site, A5SS: alternative 5′ splice site, MXE: mutually exclusive exon, RI: retained intron, SE: skipped exon.

**
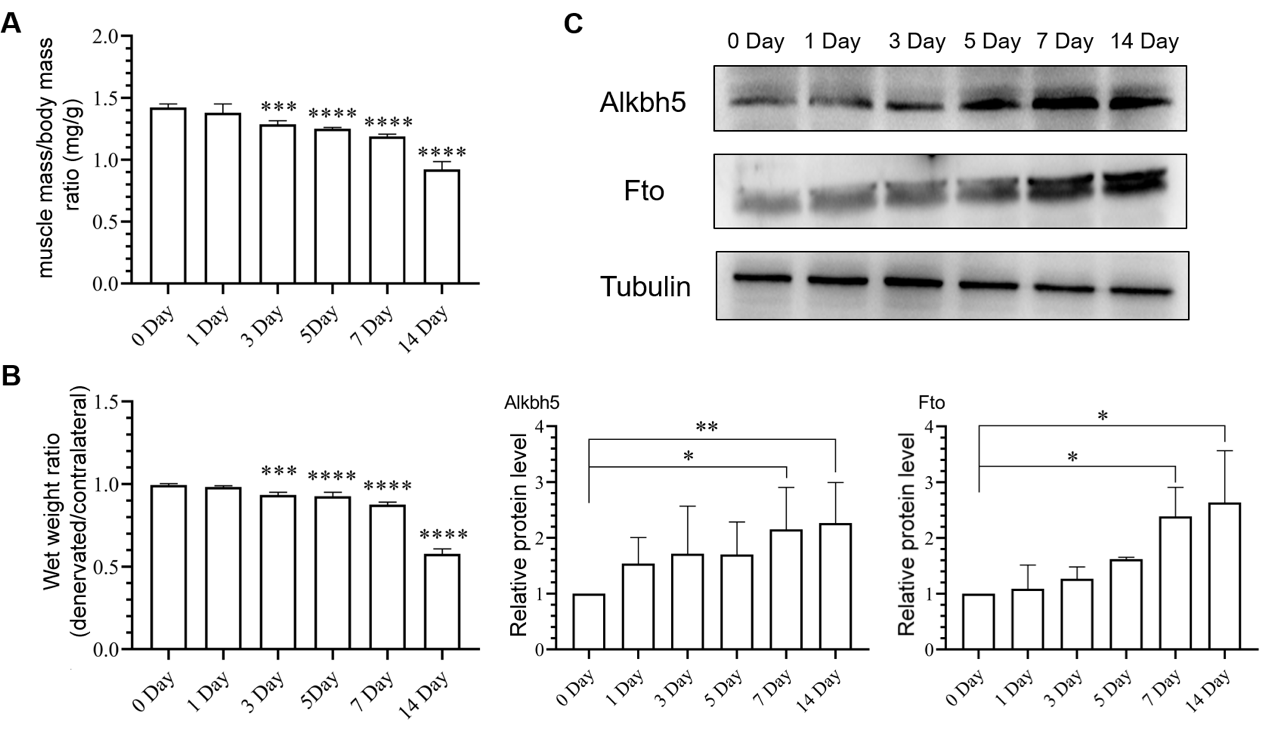
Sup-Figure 3. Changes in demethylase expression during denervated muscle atrophy**

(A) Muscle mass/body mass ratio in different time points. (B) Wet weight ratio in different time points. (C) Expression of Alkbh5 and Fto in target muscles. Data were expressed as mean±SD, n=5, *, *P*＜0.05 versus Ctrl group;**, *P*<0.01 versus Ctrl group.

**Sup-Table 1.** List of PCR primer sequences.

| gene | Direction | Sequence (5’ to 3’) |
| --- | --- | --- |
| mettl3 | F | AGGAGTGGGAGTCGCAGGAATG |
|  | R | GTCAGAAGTAGCAGAACAGCACCTC |
| mettl14 | F | GCACGCTTAGGAGGAAGAACTGAC |
|  | R | GAAGACAAGGTGAGAGCAGGCAAG |
| wtap | F | TGAGATCAACAATGGTAGACCC |
|  | R | TTGGTTCTCCTGGATAAGCATT |
| fto | F | GCAGCTGAAATACCCTAAACTG |
|  | R | AGTCTGGTGTTCAAGTACTTGT |
| alkbh5 | F | TGTTCTTGGCTTTCCTCCTTGATGG |
|  | R | TGTCTCTACTGGCTACTCTGGTGTG |
| virma | F | CCCGGGAGTACGAGCCC |
|  | R | TCATCATATTCCAGGCTTCCCA |
| 18s | F | GGACACGGACAGGATTGACAGATTG |
|  | R | TAACCAGACAAATCGCTCCACCAAC |
